# Nitrated Fatty Acids Reverse Cigarette Smoke-Induced Alveolar Macrophage Activation and Inhibit Protease Activity via Electrophilic S-Alkylation

**DOI:** 10.1371/journal.pone.0153336

**Published:** 2016-04-27

**Authors:** Aravind T. Reddy, Sowmya P. Lakshmi, Ramamohan R. Muchumarri, Raju C. Reddy

**Affiliations:** 1 Department of Medicine, Division of Pulmonary, Allergy and Critical Care Medicine, University of Pittsburgh School of Medicine, Pittsburgh, PA, 15213, United States of America; 2 Veterans Affairs Pittsburgh Healthcare System, Pittsburgh, PA, 15240, United States of America; Augusta University, UNITED STATES

## Abstract

Nitrated fatty acids (NFAs), endogenous products of nonenzymatic reactions of NO-derived reactive nitrogen species with unsaturated fatty acids, exhibit substantial anti-inflammatory activities. They are both reversible electrophiles and peroxisome proliferator-activated receptor γ (PPARγ) agonists, but the physiological implications of their electrophilic activity are poorly understood. We tested their effects on inflammatory and emphysema-related biomarkers in alveolar macrophages (AMs) of smoke-exposed mice. NFA (10-nitro-oleic acid or 12-nitrolinoleic acid) treatment downregulated expression and activity of the inflammatory transcription factor NF-κB while upregulating those of PPARγ. It also downregulated production of inflammatory cytokines and chemokines and of the protease cathepsin S (Cat S), a key mediator of emphysematous septal destruction. Cat S downregulation was accompanied by decreased AM elastolytic activity, a major mechanism of septal destruction. NFAs downregulated both Cat S expression and activity in AMs of wild-type mice, but only inhibited its activity in AMs of PPARγ knockout mice, pointing to a PPARγ-independent mechanism of enzyme inhibition. We hypothesized that this mechanism was electrophilic S-alkylation of target Cat S cysteines, and found that NFAs bind directly to Cat S following treatment of intact AMs and, as suggested by *in silico* modeling and calculation of relevant parameters, elicit S-alkylation of Cys25 when incubated with purified Cat S. These results demonstrate that NFAs’ electrophilic activity, in addition to their role as PPARγ agonists, underlies their protective effects in chronic obstructive pulmonary disease (COPD) and support their therapeutic potential in this disease.

## Introduction

Chronic obstructive pulmonary disease (COPD) is the third leading cause of death in the US [1], characterized by chronic inflammation of small airways and destruction of alveolar septa with consequent emphysema [2]. Such septal destruction reduces the surface area available for pulmonary O_2_ and CO_2_ exchange, and combines with airway inflammation and mucus production to impair respiratory mechanics. Because COPD is progressive and often fatal, and no effective therapies are available to impede its course, identification of new potential therapeutic targets is needed.

Among the many cell types that contribute to COPD pathophysiology, alveolar macrophages (AMs) are especially critical [3]. They are directly exposed to cigarette smoke, the major risk factor for COPD, and other noxious airborne agents. Such agents activate AMs to produce pro-inflammatory cytokines that activate other cells, and chemokines that attract neutrophils and T lymphocytes, prominent in COPD-associated inflammation. Such activated and recruited cells likewise secrete mediators that further activate AMs in a vicious positive-feedback cycle. Yet AMs also exert an inflammation-dampening influence by phagocytosing apoptotic neutrophils and epithelial cells that, if allowed to become necrotic, would further feed the inflammatory cycle. Such phagocytotic activity of AMs is reduced in COPD, thereby exacerbating pathogenesis [4].

AMs are also the primary sources of elastin-degrading proteases that largely drive septal destruction [5]. Most recent attention has focused on the Zn-containing matrix metalloproteinases (MMPs), but cathepsins, including cathepsins K, L, and S, are also important mediators of septal degradation [6]. Production of inflammation–associated oxidants by AMs can also contribute to septal destruction.

Inflammation-associated oxidants include NO and derived reactive nitrogen species that react nonenzymatically with unsaturated fatty acids to produce nitrated fatty acids (NFAs), including 10-nitro-oleic acid (OA-NO_2_) and 12-nitrolinoleic acid (LNO_2_) [7], the most prevalent NO reaction products in the human bloodstream [8]. NFAs are potentially important endogenous modulators of inflammatory processes [9–11], acting at least in part as agonists of the nuclear hormone receptor peroxisome proliferator activated receptor-γ (PPARγ) [12]. They are also reversible electrophiles [13]. As such, they can react with thiol groups including those of cysteine residues within proteins to produce an S-alkyl bond that is cleavable (thus reversible) by reaction with free intracellular thiols such as glutathione (GSH). It has long been observed that specific cysteines are preferentially alkylated, but the factors that render such specific cysteines more readily or stably alkylated are unknown. Known cysteine targets of NFA S-alkylation include one within the ligand-binding site of PPARγ that contributes significantly to agonist potency [14].

Reversible Cys S-alkylation provides a unique regulatory mechanism for biologically active proteins. For example, reversible electrophiles regulate oxidative stress by reversibly S-alkylating the inhibitory protein Keap-1, allowing activation of the antioxidant transcription factor Nrf2 [15]. NFAs may also alleviate inflammation by S-alkylating the pro-inflammatory transcription factor NF-κB [10]. No such influence of reversible electrophiles in general or NFAs in particular has been recognized in the pathophysiology of COPD. We hypothesized that anti-inflammatory and related actions of NFAs may mitigate COPD pathogenesis, including secretion of the proteases that mediate septal destruction, and tested this idea and the mechanisms involved in AMs from smoke-exposed mice.

## Materials and Methods

### Animals

C57BL/6 and Tie2 Cre-PPARγ^flox/flox^ mice [16] were expanded from breeding pairs. Mice were housed in microisolator cages under specific pathogen-free conditions and fed autoclaved food. Male mice aged 6–8 weeks (20–25 g) were used in all experiments. All studies were performed according to protocols reviewed and approved by the VA Pittsburgh Healthcare System Institutional Animal Care and Use Committee.

### Smoke Exposure

Mice were exposed to cage-air cigarette smoke (CS) or to filtered air for 14 days. CS was generated by burning five 3RF4 research cigarettes (Tobacco Research Institute, University of Kentucky, Lexington, KY) according to the Federal Trade Commission (FTC) protocol, each puff being of 2-second duration and 35-ml volume, in an automated TE-10 smoking machine (Teague Enterprises, Davis, CA). The machine was adjusted to produce 89% sidestream and 11% mainstream smoke. The chamber atmosphere was monitored to maintain TPM at 250 mg/m^3^. Twenty-four hours following the last exposure, mice were euthanized and BAL fluid and lungs were collected for further analysis.

### BAL Fluid Collection, Cell Count and AM Isolation from Mice

BAL fluid was collected by flushing 3 × 1 ml of PBS containing 0.1 mM EDTA into the lung via a tracheal cannula. The pooled BAL fluid was centrifuged at 500 × g at 4°C for 5 min. Pelleted cells were then resuspended in 1 ml of PBS. Total cell number was counted by hemocytometer and differential cell count was performed using cytospin preparations stained with Diff-Quik (Siemens, Newark, DE). AMs were isolated as described previously [17].

### Measurement of Cytokine, Chemokine and Cathepsin S Levels

Cell culture medium was collected and stored at –80°C. Levels of TNF-α and KC were measured using ELISA kits (R&D Systems, Minneapolis, MN) according to the manufacturer’s instructions. Cat S levels in media samples were measured using an ELISA kit (MyBioSource, Inc, San Diego, CA) according to the manufacturer’s instructions.

### Western Blotting

Total protein extracts were prepared and Western blotting was performed as described previously [18]. Primary antibodies against NF-κB p65 (372), PPARγ (7196), lamin B1 (20682), biotin (57636), Cat S (6505), and β-actin (1616) were from Santa Cruz Biotechnology (Santa Cruz, CA). The secondary antibodies donkey anti-mouse IR-680RD (926–68072), donkey anti-rabbit IR-680RD (925–68073), donkey anti-goat IR-680RD (926–68074), goat anti-rabbit IR-800CW (925–32211), and goat anti-mouse IR-800CW (926–32210), were from LI-COR (Lincoln, NE). The infrared signal was detected using an Odyssey Infrared Imager (LI-COR).

### Immunoprecipitation

Total protein extracts were immunoprecipitated using the Dynabeads Protein G Immunoprecipitation kit (Invitrogen, Carlsbad, CA). Antibodies were bound to Dynabeads Protein G, and the Dynabeads-Ab complex was used to precipitate target proteins from the total protein extracts. Precipitates were washed to remove unbound proteins and complexes were eluted. All samples were separated by electrophoresis on SDS-polyacrylamide gels, transferred to membranes, and Western blotting was performed.

### EMSA

Nuclear proteins (5 μg) were incubated with 2.5 nM infrared dye (IRDye) 700 end-labeled double stranded consensus oligonucleotides for NF-κB or PPARγ (Table 1) in 10× binding buffer (100 mM Tris, 500 mM KCl, 10 mM DTT; pH 7.5), poly dIdC (1 μg/μl in 10 mM Tris, 1 mM EDTA), 25 mM DTT and 2.5% Tween 20. Samples were then separated on 5% non-denaturing polyacrylamide gels in 1× Tris-Borate EDTA buffer (130 mM Tris, pH 8.3, 45 mM boric acid, 2.5 mM EDTA). The infrared signal was detected using an Odyssey Infrared Imager (LI-COR).

**Table 1:** Consensus oligonucleotides employed.

Binding site	Prime	Sequence	End labelling
κB Site	F	5′-AGTTGAGGGGACTTTCCCAGGC-3′	IRDye 700
	R	3′-TCAACTCCCCTGAAAGGGTCCG-5′	IRDye 700
PPRE Site	F	5′-AGACAAGTCAGAGGCCACGGT-3′	IRDye 700
	R	3′-TCTGTTCAGTCTCCGGTGCCA-5′	IRDye 700
Nonspecific Control	F	5′-AGACTGGGGCTGGAGTGCGGTT-3′	IRDye 700
	R	3′-TCTGACCCCGACCTCACGCCAA-5′	IRDye 700

### Monocyte Chemotaxis Assay

Mouse blood monocytes were isolated using an EasySep mouse monocyte enrichment kit (StemCell Technologies Inc, Vancouver, BC, Canada). Briefly, leukocyte pools from blood were incubated with an antibody mixture against non-monocytes followed by a biotin selection mixture and magnetic particles. Labeled cells were removed using DynaMag-2 magnet (Invitrogen), resulting in highly significant enrichment of monocytes in the remaining cell preparation.

Blind-well chemotaxis chambers separated with 8-mm pore polycarbonate membrane (Neuro Probe Inc., Gaithersburg, MD) were used to assess migration of monocytes toward cell culture media. The upper wells contained monocytes isolated as described above; the lower wells were loaded with culture media supernatant from macrophages isolated from mice exposed to cigarette smoke for 14 days and then treated either with vehicle or with OA-NO_2_ for 6 h. The chambers were incubated at 37°C for 1 h, after which the membranes were fixed and stained with Diff-Quik stains. The membranes were retrieved and mounted on glass slides, covered with a coverslip, and examined under a microscope. The number of cells that migrated through the membrane into the lower chamber and cells that were attached on the lower side of the membrane were counted and transmigration was expressed as % of monocytes added in upper chamber.

### Elastolytic Activity Assay

Intracellular and extracellular elastin degradation studies were performed using soluble and insoluble fluorescein (FITC) labeled elastin respectively (Elastin Products Company, Inc, Owensville, MO). To determine extracellular elastolytic activity insoluble FITC-elastin was added to culture media or BAL fluid and treated as indicated. After incubation, insoluble elastin was removed by centrifugation and fluorescence in supernatants was determined using excitation at 490 nm and emission at 520 nm. Intracellular elastolytic activity was determined by incubating AMs treated as indicated above with soluble FITC-elastin. After incubation cells were washed and mounted with Vectashield mounting medium with DAPI (Vector Laboratories, Burlingame, CA). The slides were viewed by an Olympus Fluoview FV1000 confocal microscope (Olympus, Center Valley, PA) using a 60× objective lens along with Fluoview confocal software (FV10-ASW v1.7, Olympus).

### ChIP Assay

The ChIP assay was performed using SimpleChIP Enzymatic Chromatin Immunoprecipitation kit with magnetic beads (Cell Signaling Technology, Beverly, MA, USA). Briefly, cellular chromatin was crosslinked with 1% formaldehyde for 10 min at room temperature, the crosslinking was stopped with 0.125 M glycine, and cells were washed twice with ice cold PBS. Nuclei were pelleted and digested by micrococcal nuclease. Following sonication and centrifugation, equal amounts of sheared chromatin were incubated overnight at 4°C with antibodies, IgG as negative control, or RNA polymerase II as positive control. Protein G magnetic beads were then added and the chromatin was incubated with rotation for 2 h at 4°C. An aliquot of chromatin that was not incubated with any antibody was used as the input control sample. Antibody-bound protein/DNA complexes were eluted and subjected to real-time PCR as described previously [18] with specific primers for Ctss (Cat S), CD36, and α-satellite (Table 2).

**Table 2:** Oligonucleotide primers employed.

Gene		Primer Sequence	Tm (°C)	Amplicon Size (bp)
CD36 Promoter	F	5′-AATGCTTTATTCCTCCTTGTTTCC-3′	62	96
	R	5′-TGCTAGAAAGGAAGTAGCTTCAG-3′	62	
Cat S Promoter	F	5′-GGCTCTTCTTGATGGCTTACT-3′	62	**77**
	R	5′-AGCTAGTACAGTCACCTCTAGTC-3′	62	
α-satellite		Cat #4486, Cell Signaling Technology		

### Molecular Modeling and Computer Simulations of Binding of OA-NO_2_ with Cat S

Molecular modeling and covalent docking analysis was carried out as described previously [19]. The X-ray structure of human Cat S {PDB ID 1NPZ; [20]} and its C25S mutant {PDB ID 1GLO; [21]} were obtained from the RCSB Protein Data Bank. Covalent docking was performed with OA-NO_2_ (ligand) using Discovery Studio 2.5 (Accelrys Inc., San Diego, CA).

### Cat S Enzyme Inhibition Assay

Cat S enzyme inhibition assay was performed using the Cathepsin S Drug Discovery Kit (BML-AK431-0001; Enzo Life Sciences, Farmingdale, NY) according to the manufacturer’s instructions.

### Cat S Enzyme Activity Assay

Cat S enzyme activity in cell culture media samples was measured by incubating these samples with Cat S-specific fluorescent substrate (Ac-KQKLR-AMC; 600742, Cayman Chemical) according to the manufacturer’s instructions.

### Cat S Cys Mutants

A plasmid containing the human Cat S gene was obtained from Addgene (#11251) and subcloned into a pGEX 6P1 plasmid. Cysteine residues at positions 12, 25 and 110 or all three (12, 25 and 110) were mutated to serine using site directed mutagenesis (custom cloning core facility at Emory University, Atlanta, GA). Recombinant proteins were expressed in BL21-DE3 cells (Sigma-Aldrich, St. Louis, MO), purified using GST SpinTrap columns (GE Healthcare Life Sciences), and the GST tag was removed with PreScission Protease (GE Healthcare Life Sciences) according to the manufacturer’s instructions.

### Cat S S-alkylation

To demonstrate S-alkylation of Cat S, human recombinant Cat S (EMD Millipore Billerica, MA) and variants−Cat S WT, C12;25;110S mutant, C12S mutant, C25S mutant, and C110S mutant−were incubated with test compounds for the indicated time periods and S-alkylation was analyzed by running these samples in a SDS-PAGE gel under non-reducing conditions and performing Western blots using antibody to biotin (Santa Cruz Biotechnology).

### Electrospray Ionization-Liquid Chromatography-Mass Spectrometry/Mass Spectrometry (ESI-LC-MS/MS)

ESI-LC-MS/MS analyses were performed on a Q Exactive Hybrid Quadrupole-Orbitrap mass spectrometer (Thermo Scientific, Pittsburgh, PA). The electrospray system employed a 3.5 kV spray voltage and a capillary temperature of 260°C.

### Statistical Analysis

Data are presented as mean ± SD. Differences between groups were analyzed using an unpaired t-test or analysis of variance, followed by Bonferroni multiple comparison correction. Analyses were performed using GraphPad Prism 5.03 (GraphPad Software, La Jolla, CA). Differences with *P* < 0.05 were considered significant.

## Results

### OA-NO_2_ reduces cigarette smoke-induced AM activation in mice

**Fig 1:**
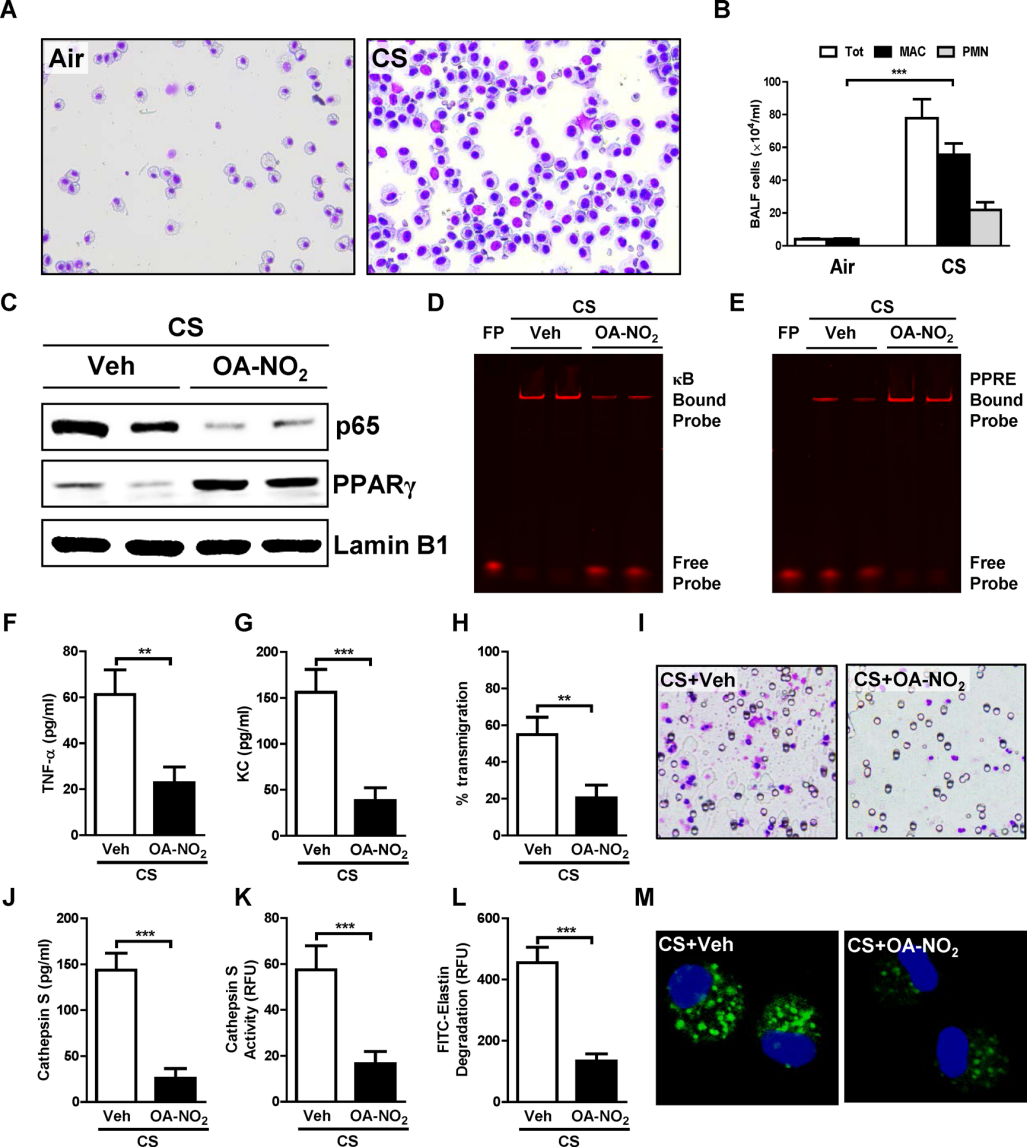
NFAs mitigate cigarette smoke-induced alveolar macrophage activation. (A, B) C57BL/6 mice were exposed to cigarette smoke (CS) or to filtered-air for 14 days. Twenty-four h after the final exposure, BAL samples were collected. (A) Photomicrographs (40× objective lens) of Diff-Quik-stained cells and (B) total (Tot), neutrophil (PMN), and macrophage (MAC) counts in BAL fluid from the indicated treatment groups. (C-M) Alveolar macrophages (AMs) were isolated from BAL fluid of mice exposed to CS as described above and were then treated with 1 μM OA-NO_2_ or vehicle (Veh) for 6 h. (C) Western blots for PPARγ and p65 in nuclear extracts. (D, E) DNA-binding activity of (D) NF-κB, and (E) PPARγ, in OA-NO_2_ or Veh treated AM extracts measured by EMSA using IRDye oligonucleotides. (F, G) Release of (F) TNFα. and (G) KC. (H, I) Mouse monocytes were isolated and transmigration was assayed using a chemotaxis chamber in which they were separated by an 8-mm pore polycarbonate membrane from chemoattractant culture media from the above treatment groups. (H) Percentage transmigration, and (I) photomicrographs (40× objective lens) of the lower side of the polycarbonate membrane. (J) Cat S expression and (K) activity in culture media from above treatment groups. (L, M) AMs were collected and treated as indicated above, then incubated with FITC-elastin for further 1 h to determine (L) extracellular and (M) intracellular elastolytic activity (60× objective lens). Data are representative of two to three independent experiments with cells obtained from *n* = 14–18 mice/group (cells from 3 mice pooled together). **P < 0.01, ***P < 0.001.

Considering the pivotal role of AMs in the response to smoke inhalation and COPD pathogenesis, we tested the effects of NFAs on AM activities relevant to inflammation and septal destruction in a mouse model of COPD. We exposed mice to CS delivered via cage air for 14 days. Smoke exposure greatly increased the numbers of macrophages in BAL fluid, with lesser increases in the number of neutrophils (Fig 1A and 1B). Because AMs represented the majority of inflammatory cells found in BAL fluid of smoke-exposed mice, we assessed them further.

*In vitro* OA-NO_2_ treatment (no toxicity was observed, S1 Fig) of AMs isolated from BAL fluid of smoke-exposed mice reduced both nuclear expression and DNA-binding activity of the pro-inflammatory transcription factor NF-κB (Fig 1C and 1D), but increased those of the anti-inflammatory nuclear hormone receptor PPARγ (Fig 1C and 1E). Based on these changes in activity of pro- and anti-inflammatory transcriptional regulators, we predicted that expression of pro-inflammatory genes would be reduced, and indeed found that secretion of TNF-α (Fig 1F) and KC (Fig 1G) by AMs was suppressed. Secretion of chemokines was also reduced, as seen using a monocyte transmigration assay (Fig 1H and 1I). Assessing emphysema-causing protease secretion and elastin degradation, we found that OA-NO_2_ treatment markedly reduced secreted Cat S levels and activity in culture medium (Fig 1J and 1K). Degradation of elastin by culture medium of OA-NO_2_-treated AMs (Fig 1L) and intracellular elastin degradation by whole AMs (Fig 1M) were similarly reduced. Thus, OA-NO_2_ suppressed both the inflammatory and proteolytic activities induced by smoke exposure in AMs. As these cells play a prominent role in COPD pathophysiology [3], these effects carry significant implications for modulation of this disease.

### OA-NO_2_ Effects on Cathepsin S Expression, but not Activity, are PPARγ-dependent

**Fig 2:**
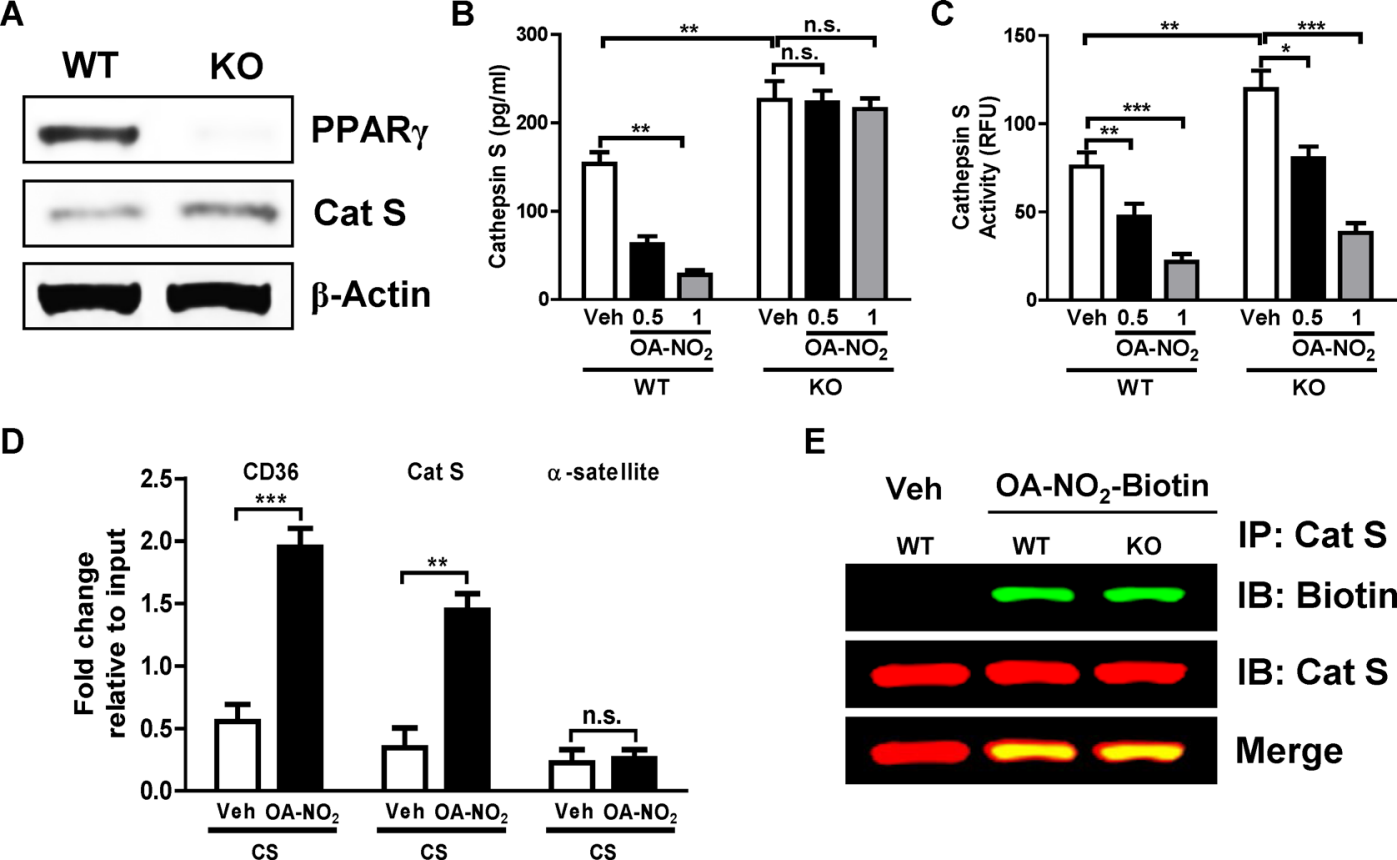
NFAs’ inhibition of Cat S expression but not activity, is PPARγ-dependent. (A) Western blots for PPARγ and Cat S in whole cell extracts from AMs isolated from BAL fluid of C57BL/6 (WT) and Tie2 Cre-PPARγ^flox^/^flox^ (KO) mice. (B, C) Mice were exposed to cigarette smoke (CS) for 14 days, AMs were isolated and then treated with 0.5 and 1 μM OA-NO_2_ or Veh for 6 h. (B) Cat S expression and (C) activity in culture media. (D) Following AM isolation and OA-NO_2_ (1 μM) treatment as above, chromatin was crosslinked and immunoprecipitated with PPARγ antibody; the antibody-bound DNA-protein complexes were then subjected to real-time PCR with primers specific for PPRE sites in Cat S and CD36 promoter regions, α-satellite was used as control. (E) As above, AMs were collected from CS-exposed mice and treated with 5 μM biotin-labeled OA-NO_2_ or Veh for 6 h. Total protein extracts were prepared, Cat S was immunoprecipitated and Western blotting was performed with anti-biotin and -Cat S antibodies. Data are representative of two to three independent experiments with cells obtained from *n* = 14–18 mice/group (cells from 3 mice pooled together). *P < 0.05, **P < 0.01, ***P < 0.001, n.s. = non-significant.

As NFAs are known to act both as reversible electrophiles and via PPARγ activation, we tested which of these mechanisms was responsible for the observed reductions in Cat S expression and activity using AMs from wild-type (WT) and PPARγ knockout (KO) mice following 14-day *in vivo* smoke exposure and 6-h *in vitro* exposure to OA-NO_2_ or vehicle. Absence of PPARγ in AMs from KO mice was confirmed by Western blotting, which also revealed modest increases in Cat S (Fig 2A). OA-NO_2_ treatment reduced Cat S expression in AMs from WT but not PPARγ KO mice (Fig 2B), indicating that NFA-induced suppression of Cat S expression requires PPARγ activation. In contrast, NFAs reduced Cat S activity in culture media of AMs to approximately the same extent irrespective of the presence or absence of PPARγ (Fig 2C). Therefore, inhibition of Cat S activity does not depend on PPARγ, and we thus hypothesized it reflects S-alkylation by NFAs. Supporting this hypothesis, we found that NFA bound directly to Cat S following incubation of Cat S with biotin-labeled OA-NO_2_, immunoprecipitation, and Western blotting using anti-biotin antibody (Fig 2E). Conversely, supporting a PPARγ-dependent mechanism of NFA effects on Cat S expression, we found by chromatin immunoprecipitation (ChIP) that OA-NO_2_ treatment increased PPARγ binding to both the *Cd36* promoter, which is known to be upregulated by PPARγ activation, and also the *Ctss* (Cat S) promoter (Fig 2D). This was consistent with prior findings that under certain circumstances binding of PPARγ to DNA inhibits rather than stimulates transcription [22]. Thus, the observed NFA effects on Cat S expression and activity were mediated by distinct, complementary mechanisms in which electrophilic addition plays a prominent role.

### OA-NO_2_ Inhibits Cathepsin S by S-alkylating Cys25

**Fig 3:**
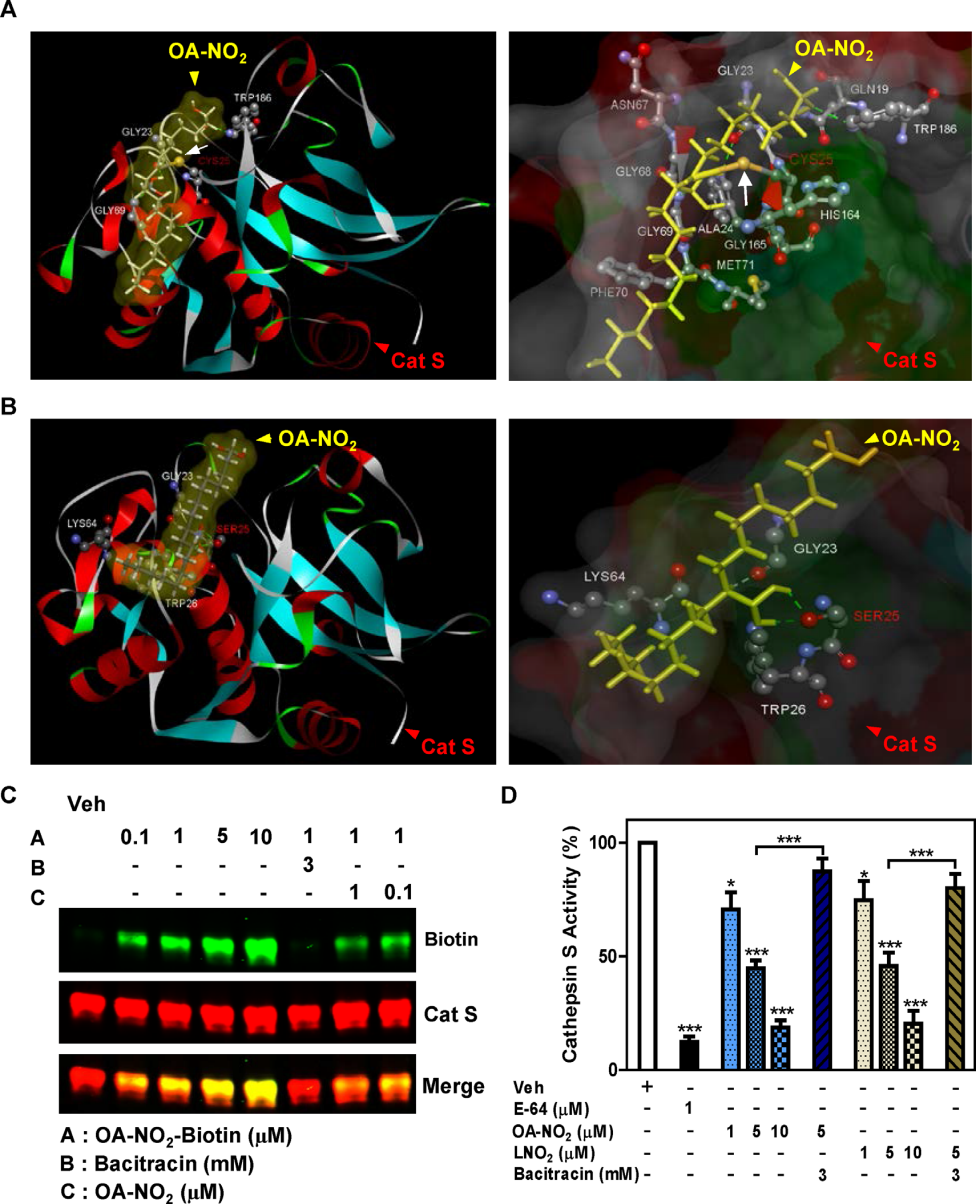
NFAs inhibit Cat S enzymatic activity by S-alkylation. (A-C) Binding of OA-NO_2_ to Cat S was modeled *in silico* and evaluated *in vitro*. (A) Schematic representation showing covalent interaction of OA-NO_2_ with Cat S Cys25 residue. (B) Schematic representation showing absence of any covalent interaction of OA-NO_2_ with Cat S Cys25Ser mutation. (*Left panels*) Cat S is shown as a flat ribbon colored according to secondary structure; OA-NO_2_ (yellow) is shown as a stick model and interacting amino acids are shown in scaled ball-and-stick representation. (*Right panels*) Cat S is shown with interacting amino acid residues as a scaled ball-and-stick model with labels and OA-NO_2_ (yellow) as a stick model. In both figures the covalent bond is indicated by *white arrow* and hydrogen bonds are indicated by *green dotted lines*. (C) Human recombinant Cat S in active form (200 ng) was incubated for 30 min with the indicated compounds. Following incubation, S-alkylation of Cat S by biotin-labeled OA-NO_2_ in test samples was assessed by Western blotting under non-reducing conditions. (D) Human recombinant Cat S in active form (6 μU/μl) was incubated for 1 h with the indicated compounds and enzyme activity determined by cleavage of fluorescent substrate peptide. Data are representative of three independent experiments with *n* = 4/group. *P < 0.5, ***P < 0.001.

We next sought to specifically identify the site of NFA-Cat S binding and the type of bond involved. Molecular docking studies revealed covalent bond formation between the electrophilic carbon of OA-NO_2_ and sulfur atom Cys25 of Cat S (Fig 3A). No covalent bond formation was seen when Cat S with Cys25 to Ser mutated was used, as the oxygen in serine is not sufficiently nucleophilic to bond with the NFA’s electrophilic carbon (Fig 3B).

We confirmed that NFA binds to Cat S protein by incubating recombinant human Cat S *in vitro* with biotin-labeled OA-NO_2_ followed by Western blotting (Fig 3C). Incubation with either OA-NO_2_ or LNO_2_ dose-dependently inhibited human Cat S activity, and at the highest dose tested exhibited inhibition similar to that seen with the classic Cat S inhibitor E-64 (Fig 3D). This inhibition was blocked by the thiol exchange inhibitor bacitracin, thus confirming S-alkylation as the mechanism of inhibition.

**Fig 4:**
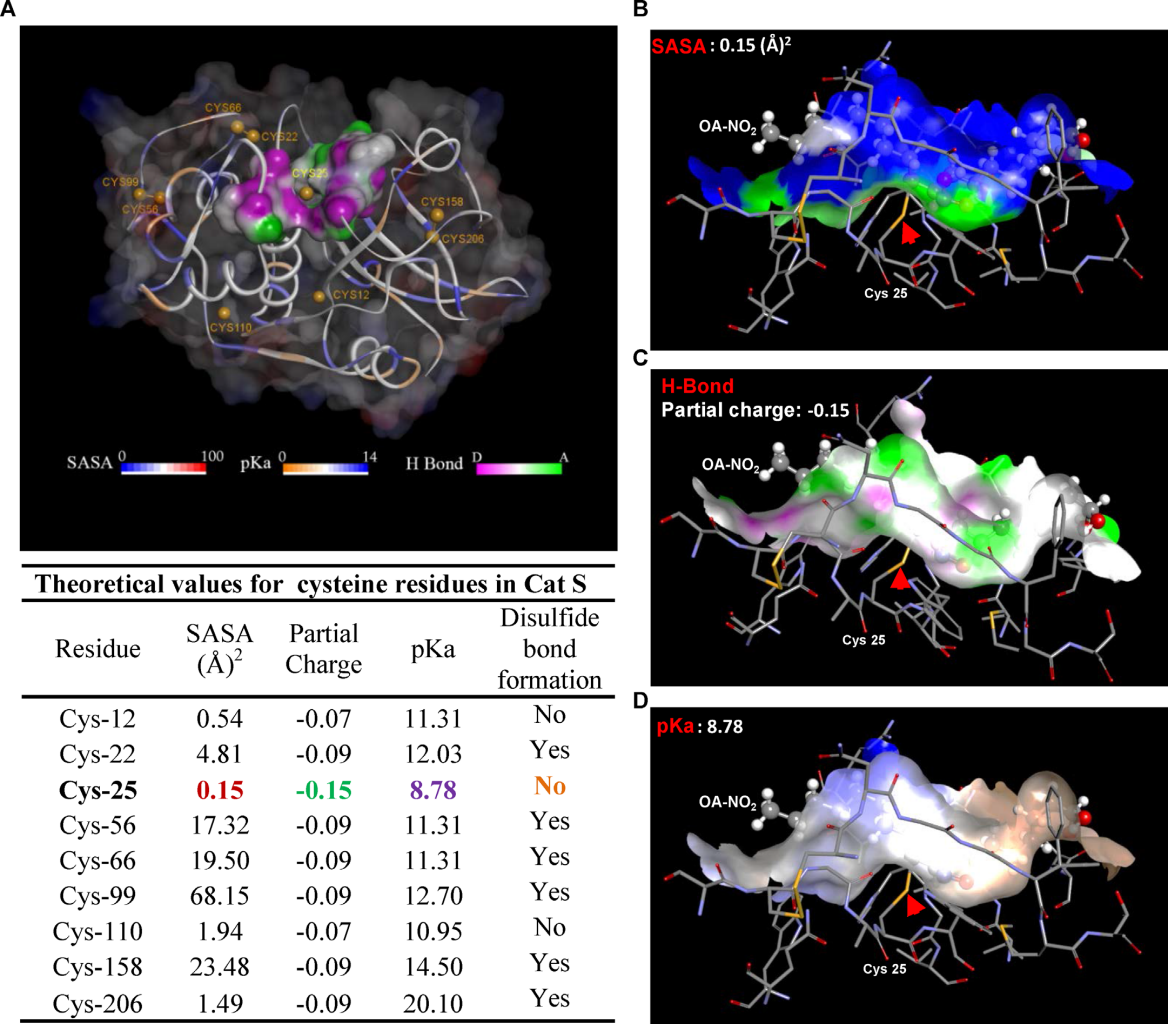
*In silico* modeling shows Cat S Cys25 is the preferred target for NFA S-alkylation. *In silico* modeling of Cat S showing potential for electrophilic S-alkylation. (A) Cysteine residues are indicated by yellow spheres and disulfide bonds by short yellow lines. Each residue is colored according to its calculated pK_a_. The protein surface is colored to indicate solvent accessibility and the surface of the potential OA-NO_2_ binding pocket is colored according to its H-bonding potential. The bar scales indicate the values corresponding to each color used. Numerical values of these parameters are shown in the accompanying table. (B-D) *In silico* models of Cat S with OA-NO_2_ bound to Cys25. Protein surface is color coded to indicate: (B) solvent accessible surface area (SASA); (C) potential for H-bond formation; (D) pKa of OA-NO_2_–Cat S binding pocket. OA-NO_2_ is rendered as a scaled ball-and-stick model and Cat S as a stick model. The covalent bond between OA-NO_2_ and Cys25 is indicated by a red arrow.

We next sought to specifically identify the site of NFA-Cat S binding and the type of bond involved. We modeled Cat S *in silico* (Fig 4) to 1) determine the presence or absence of disulfide bonding for each Cys residue and the S atom’s partial positive charge (Fig 4A); and 2) calculate solvent accessibility (Fig 4B), H-bonding potential (Fig 4C), and pKa (Fig 4D) for each of its cysteine residues. The results suggested that Cys25 is the most nucleophilic and thus the most likely site of S-alkylation. This conclusion was supported by *in silico* modeling of OA-NO_2_ bonded to Cys25, indicating appropriate solvent accessibility and the presence of stabilizing H-bonds (Fig 4B–4D).

**Fig 5:**
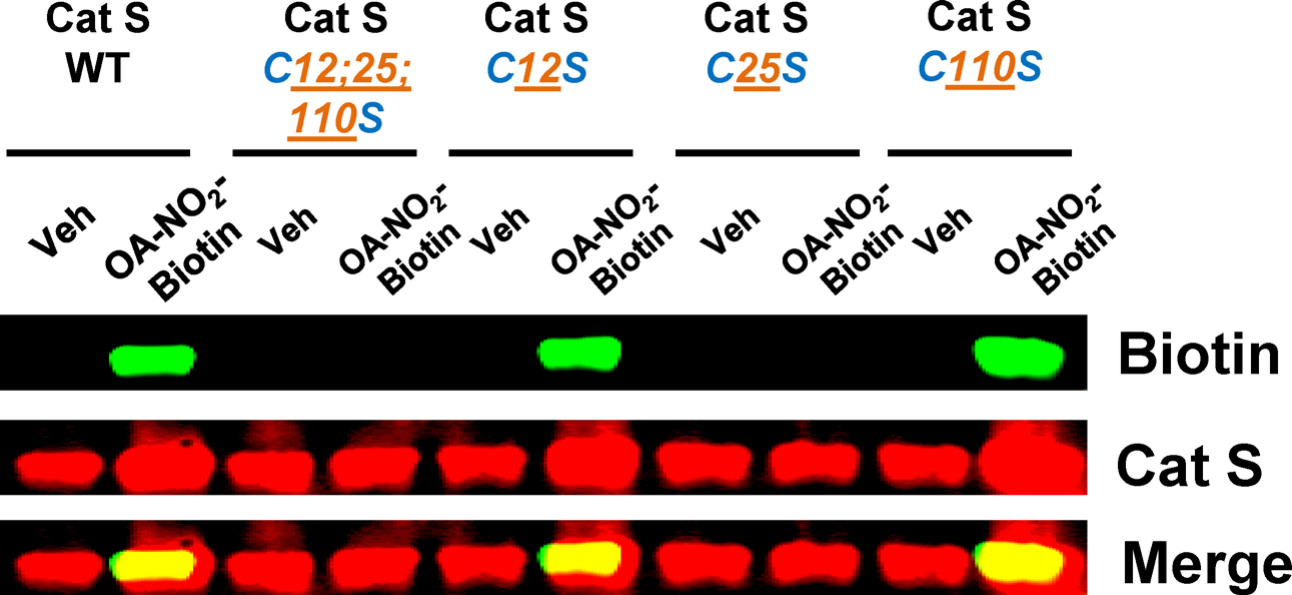
NFAs S-alkylate Cat S specifically at Cys 25. Human recombinant Cat S WT and Cys mutants C12;25;110S, C12S, C25S, C110S (200 ng) were incubated for 30 min with biotin-labeled OA-NO_2_ (1 μM). Following incubation, S-alkylation of Cat S by biotin-labeled OA-NO_2_ was assessed by Western blotting under non-reducing conditions. Data are representative of two independent experiments.

To test whether Cys25 was the specific Cat S residue to which NFAs bond covalently, we incubated biotin-labeled OA-NO_2_ with WT Cat S or Cat S with Cys residues mutated to Ser [either individually at Cys12 (C12S), Cys25 (C25S), or Cys110 (C110S), or at all three positions, (C12;25;110S)]. OA-NO_2_ binding was then determined by Western blotting (Fig 5). OA-NO_2_ was bound to Cat S by S-alkylation only in Cat S WT, C12S, and C110S mutants; the interaction was absent when Cys25 was mutated to Ser (C12;25;110S and C25S). These results indicate that the S-alkylation of Cat S by OA-NO_2_ occurs specifically at Cys25.

**Fig 6:**
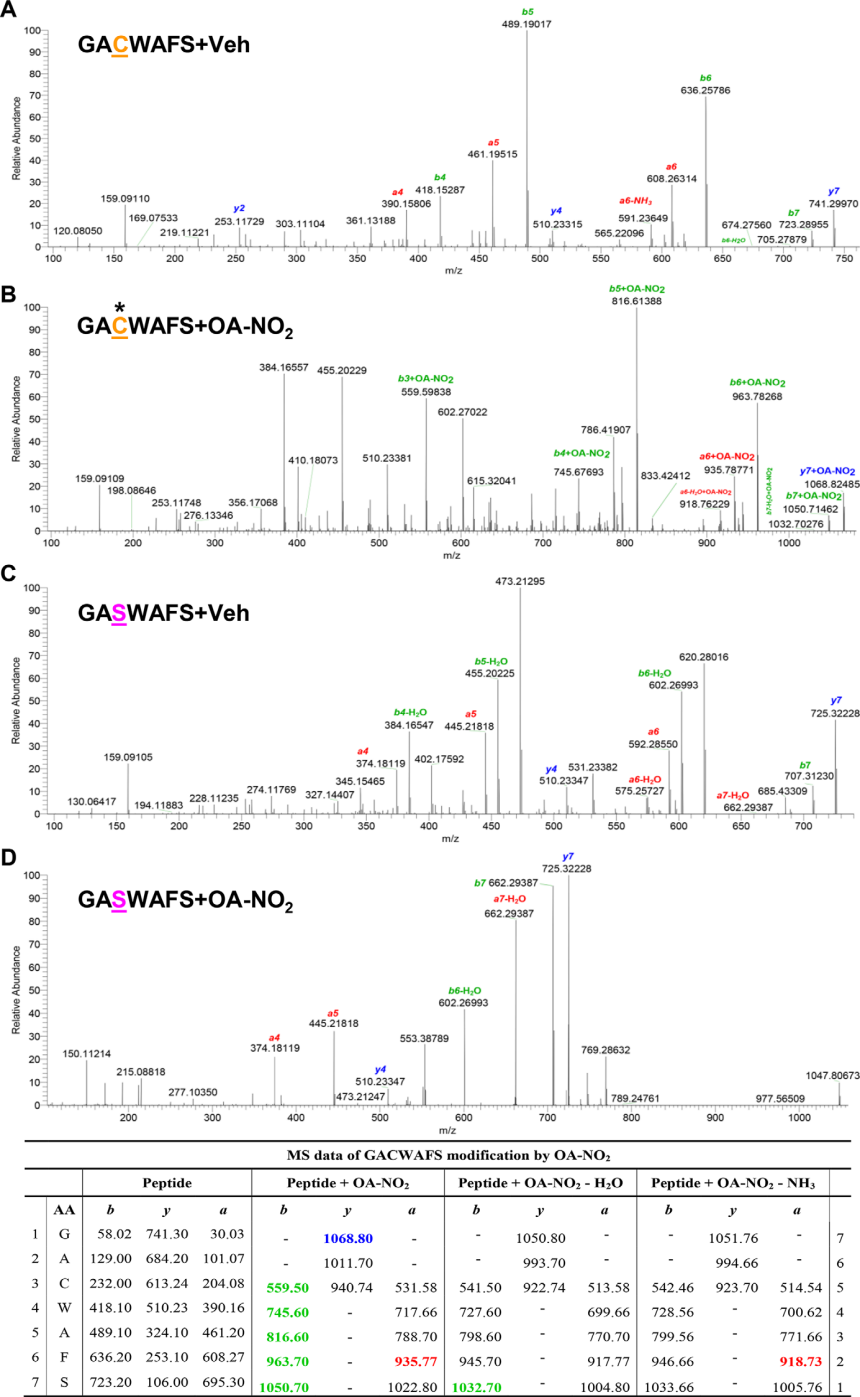
Mass spectrometric analysis of Cat S Cys25 S-alkylation. One μg of synthetic Cat S_23-29_ containing either native Cys25 (GA**C**WAFS) or Ser25 (GA**S**WAFS) was incubated *in vitro* for 30 min with (A,C) Veh or (B, D) 20 μM OA-NO_2_. Reaction mixtures were resolved by reverse phase chromatography and analyzed by electrospray ionization-tandem mass spectrometry. (A, B) The spectrum for peptide GACWAFS shows *B*, S-alkylation of OA-NO_2_ on the Cys residue but *A*, no alkylation by Veh. (C, D) Absence of S-alkylation of OA-NO_2_ with peptide GA**S**WAFS where Cys is replaced by a Ser residue. *Table* shows *m/z* of fragment ions from peptide GA**C**WAFS, with detected ions highlighted in color. Column AA identifies the corresponding amino acids. Data are representative of three independent experiments.

To further confirm the covalent interaction we incubated OA-NO_2_ (or vehicle) with a synthetic Cat S peptide (Cat S23-29) containing either the native Cys25 (sequence GA**C**WAFS) or a Cys25Ser substitution (GA**S**WAFS), and analyzed the products by mass spectrometry, which indicated that NFA was S-alkylated to the Cys25-containing fragment (Fig 6B and table). As predicted, Cys25Ser mutation abolished NFA S-alkylation (Fig 6D and table) and no addition was seen in the absence of NFA (Fig 6A and 6C and table). Our initial prediction, based on the theoretical calculations shown in Fig 4, that Cys25 would be the alkylated residue was thus confirmed.

## Discussion

We find that NFA treatment of activated AMs from CS-exposed mice downregulates expression and activity of Cat S, a protease heavily involved in the septal destruction that leads to emphysema. Because NFAs are known PPARγ agonists [23], we tested whether PPARγ mediates their suppressive effects on Cat S, finding that NFAs downregulate both Cat S gene expression and its enzymatic activity. These actions are mechanistically distinct, however, as PPARγ deletion eliminated NFA-induced downregulation of Cat S expression but not NFA-induced inhibition of Cat S enzymatic activity. Further studies demonstrated that NFAs inhibit Cat S activity via electrophilic S-alkylation. As our data show, this reversible addition of an NFA to the Cat S protein is not random, but is directed toward a specific catalytic cysteine (Cys25) that exhibits appropriate nucleophilicity, solvent accessibility, and potential of surrounding amino acids for formation of stabilizing H-bonds. Thus, we found that NFAs act through distinct PPARγ-dependent and -independent mechanisms that nonetheless act in a dual, functionally concerted manner to reduce Cat S levels, leading to the observed decrease in AMs’ extracellular and intracellular elastolytic activity. Such a reduction in vivo would be expected to alleviate COPD severity and progression toward emphysema.

Bonnaci and colleagues similarly observed PPARγ-dependent decreases in expression of MMPs, another class of COPD-related proteases, in conjunction with PPARγ-independent effects on enzyme activity [24]. Mechanisms of the apparent MMP inhibition were not investigated, however. Reversible electrophiles, including NFAs, were found earlier to S-alkylate other regulatory proteins, including the p65 subunit of NF-κB [10], protein kinase Cς [25] and Keap-1[15, 26], but these actions have not previously been linked to mechanisms of disease pathogenesis. Indeed, despite NFAs’ recognized ability to electrophilically alkylate targeted protein cysteines via Michael addition, the physiological and pathogenetic relevance of these actions has remained largely unknown prior to our current investigations.

The biological roles of endogenous NFAs are likewise uncertain, but available evidence suggests that they may be important inflammatory modulators. They are produced by nonenzymatic reaction of unsaturated fatty acids with NO-derived reactive nitrogen species [7], production of which is upregulated during inflammation. Inflammation also upregulates cholesteryl hydrolase activity [27], which drives release of free, active NFAs from their esters. Even in the absence of inflammation, the combined concentrations of free and esterified fractions of NFAs in blood are within the range required to activate PPARγ [8]. Endogenous NFAs may also downregulate inflammation by S-alkylating NF-κB, and may alleviate inflammation-produced oxidative stress by S-alkylating Keap-1 [26]. Definitive proof of NFAs’ endogenous role has remained elusive, however, because traditional techniques are not applicable: Endogenous NFA production cannot be effectively blocked and their multiple mechanisms of action, as illustrated by our present results, render it difficult to block their effects. Indeed, it will be challenging to block S-alkylation experimentally in intact cells.

**Fig 7:**
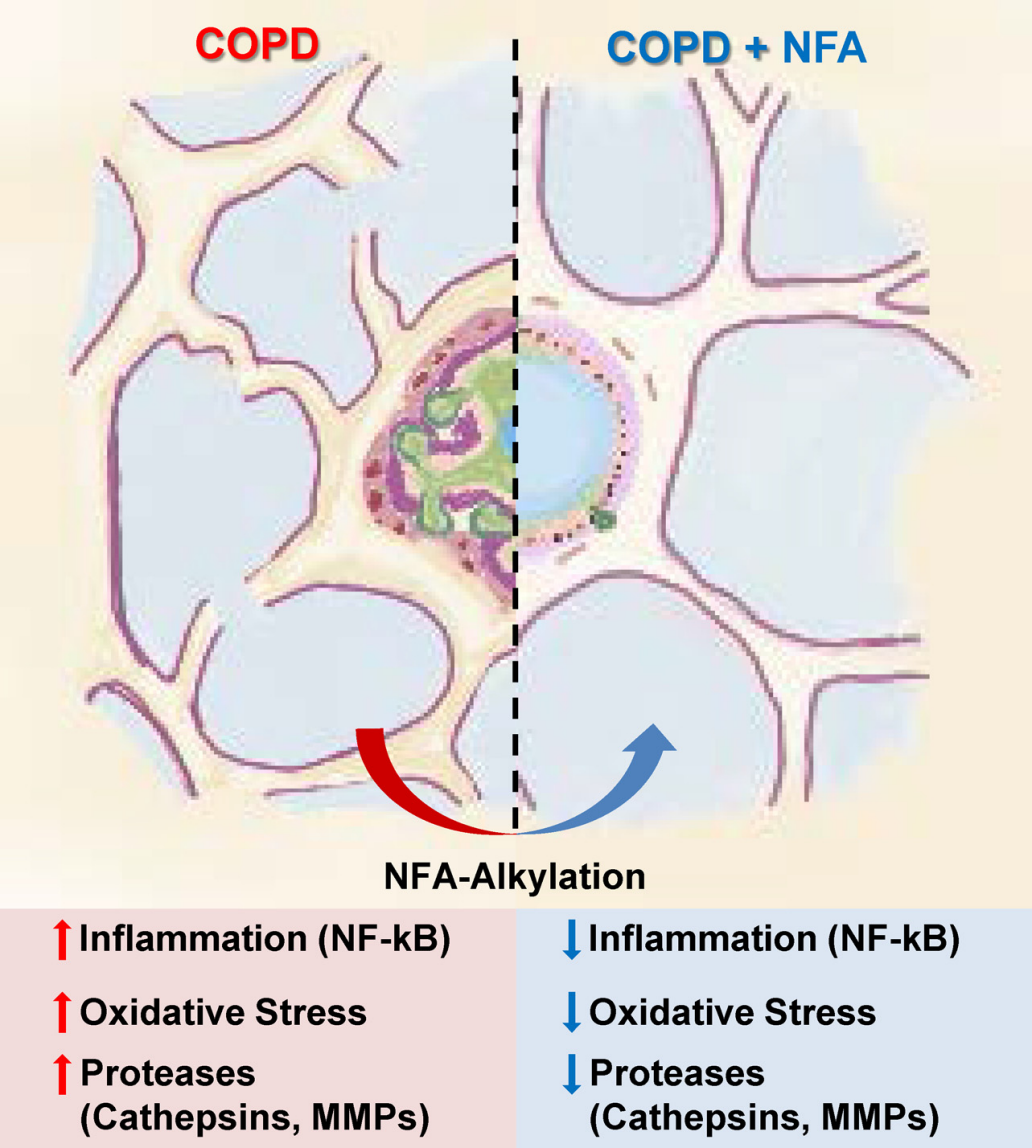
Schematic illustration of effects of S-alkylation by NFAs on COPD. Exposure to cigarette smoke induces COPD with accompanying increases in inflammation (NF-κB), oxidative stress, and release of elastolytic proteases (cathepsins and MMPs). Treatment with NFAs, acting partially through electrophilic S-alkylation of specific cysteines, reverses all these COPD-associated changes.

Beyond Cat S regulation, we found that NFAs downregulate activation of AMs obtained from smoke-exposed mice, as assessed by a variety of inflammatory markers including NF-κB activity, cytokine/chemokine production, and transmigration. AMs are pivotal contributors to inflammation, particularly in COPD [3]. Through their production of inflammatory cytokines and chemokines [28], activated AMs serve to activate and recruit other cells, complementing their own direct pro-inflammatory actions. Our finding that treating AMs with OA-NO_2_ reduces ability of their conditioned medium to recruit additional macrophages reveals a potentially powerful pathway via which NFAs can act to alleviate inflammation. As key sources of the principal elastolytic proteases, MMPs and cathepsins [5], AMs also contribute crucially to emphysema-causing septal destruction. Thus, our finding that NFAs downregulate total Cat S activity via both PPARγ- and electrophile-dependent mechanisms, while others found NFAs downregulate MMP expression [24], points to a potentially important protective role of endogenous or therapeutically-aimed NFAs in COPD (Fig 7).

In summary, our findings demonstrate that NFAs act as both electrophiles and PPARγ agonists to suppress the pathophysiology of CS-induced COPD. In particular, NFAs downregulate expression of the septum-destroying protease Cat S while specifically S-alkylating the enzyme’s catalytic Cys25, thus inhibiting its elastolytic activity and ability to promote emphysema. These results provide mechanistic insights into the part NFAs may play in modulating COPD severity, highlighting the novel role of their electrophilic activity, and support their potential therapeutic role in this disease.

## Supporting Information

S1 Fig OA-NO_2_ treatment has no toxic effects on mouse AMs. AMs were isolated and cultured as described and were treated with OA-NO_2_ (0.1, 0.5, 1, 5 and 10 μM) for 6 h. After treatment AM viability, cytotoxicity and apoptosis were assayed as indicated in *Materials and Methods*. Data are representative of two independent experiments with *n* = 3/group.
(TIFF)

S1 File Materials and Methods. **Viability, Cytotoxicity and Apoptosis.** Mouse AMs were isolated and treated as indicated with OA-NO_2_. Cell viability, cytotoxicity and apoptosis were measured according to manufacturer’s instructions using ApoTox-Glo Triplex Assay (Promega, Madison, WI). Briefly, at the end of the OA-NO_2_ treatment period 20 μl of viability/cytotoxicity reagent containing both GF-AFC substrate and bis-AAF-R110 substrate was added to all wells, briefly mixed by shaking, and incubated for 30 min at room temperature. Viability and cytotoxicity were assessed by fluorescence measured using a plate reader (VICTOR X, PerkinElmer; Waltham, MA). After measurement, 100 μl of Caspase-Glo 3/7 reagent was added to each well, briefly mixed by shaking, and incubated for 30 min at room temperature. Apoptosis was assessed by luminescence measured using a plate reader.
(DOCX)
