# Supplementary figures and images for: Nitrated Fatty Acids Reverse Cigarette Smoke-Induced Alveolar Macrophage Activation and Inhibit Protease Activity via Electrophilic S-Alkylation
Source: PLoS One. 2016 Apr 27;11(4):e0153336. doi: 10.1371/journal.pone.0153336 (PMC4847772; doi:10.1371/journal.pone.0153336)

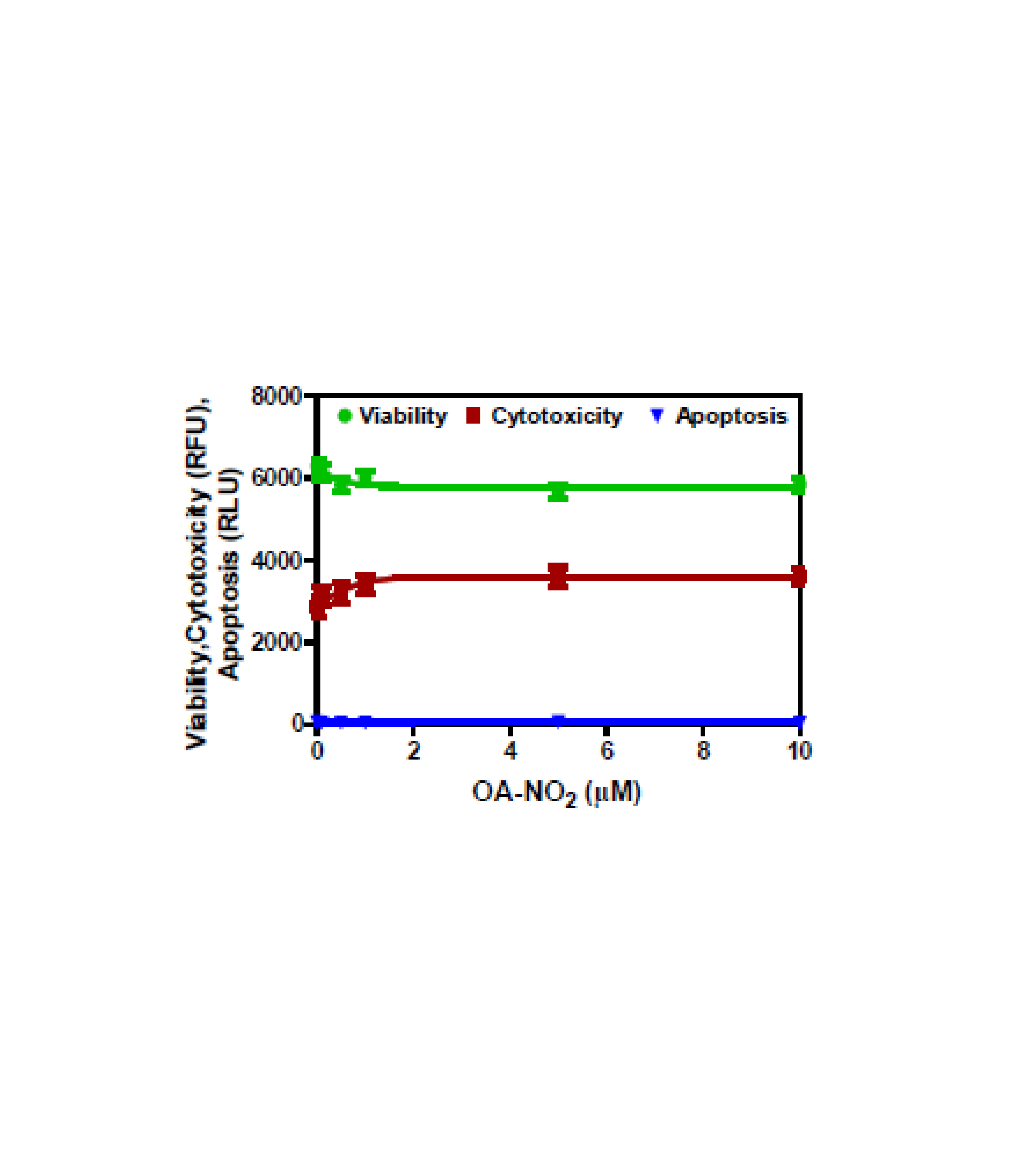

Supplement: S1 Fig — AMs were isolated and cultured as described and were treated with OA-NO2 (0.1, 0.5, 1, 5 and 10 μM) for 6 h. After treatment AM viability, cytotoxicity and apoptosis were assayed as indicated in Materials and Methods. Data are representative of two independent experiments with n = 3/group. (TIFF) [file pone.0153336.s001.tiff]
