# Supplementary material for: Nitrated Fatty Acids Reverse Cigarette Smoke-Induced Alveolar Macrophage Activation and Inhibit Protease Activity via Electrophilic S-Alkylation
Source: PLoS One. 2016 Apr 27;11(4):e0153336. doi: 10.1371/journal.pone.0153336 (PMC4847772; doi:10.1371/journal.pone.0153336)
Supplement: S1 File — Viability, Cytotoxicity and Apoptosis. Mouse AMs were isolated and treated as indicated with OA-NO2. Cell viability, cytotoxicity and apoptosis were measured according to manufacturer’s instructions using ApoTox-Glo Triplex Assay (Promega, Madison, WI). Briefly, at the end of the OA-NO2 treatment period 20 μl of viability/cytotoxicity reagent containing both GF-AFC substrate and bis-AAF-R110 substrate was added to all wells, briefly mixed by shaking, and incubated for 30 min at room temperature. Viability and cytotoxicity were assessed by fluorescence measured using a plate reader (VICTOR X, PerkinElmer; Waltham, MA). After measurement, 100 μl of Caspase-Glo 3/7 reagent was added to each well, briefly mixed by shaking, and incubated for 30 min at room temperature. Apoptosis was assessed by luminescence measured using a plate reader. (DOCX) [file pone.0153336.s002.docx]

**Supporting Information**

**Materials and Methods**

**Viability, Cytotoxicity and Apoptosis**

Mouse AMs were isolated and treated as indicated with OA-NO_2_. Cell viability, cytotoxicity and apoptosis were measured according to manufacturer’s instructions using ApoTox-Glo Triplex Assay (Promega, Madison, WI). Briefly, at the end of the OA-NO_2_ treatment period 20 μl of viability/cytotoxicity reagent containing both GF-AFC substrate and bis-AAF-R110 substrate was added to all wells, briefly mixed by shaking, and incubated for 30 min at room temperature. Viability and cytotoxicity were assessed by fluorescence measured using a plate reader (VICTOR X, PerkinElmer; Waltham, MA). After measurement, 100 μl of Caspase-Glo 3/7 reagent was added to each well, briefly mixed by shaking, and incubated for 30 min at room temperature. Apoptosis was assessed by luminescence measured using a plate reader.

**Figure Legend**

**Supporting Figure 1.** **OA-NO_2_ treatment has no toxic effects on mouse AMs**. AMs were isolated and cultured as described and were treated with OA-NO­_2_ (0.1, 0.5, 1, 5 and 10 µM) for 6 h. After treatment AM viability, cytotoxicity and apoptosis were assayed as indicated in *Materials and Methods*. Data are representative of two independent experiments with *n* = 3/group.
